# Protocol for high-content drug screening using tumor organoids on a 384-pillar plate platform

**DOI:** 10.1016/j.xpro.2025.104250

**Published:** 2025-12-01

**Authors:** Jung Sook Choi, Min Sang Lee, Myung Jin Song, Kwiwan Jeong

**Affiliations:** 1 Bio Industry Department, Gyeonggido Business & Science Accelerator, Suwon 16229, Republic of Korea

**Keywords:** Cancer, High-throughput screening, Organoids

## Abstract

Tumor organoids provide physiologically relevant three-dimensional (3D) models that overcome the limitations of two-dimensional cultures in drug screening. Here, we present a protocol for generating compact and reproducible ovarian cancer organoids using SKOV-3 cells embedded in growth-factor-reduced Matrigel and dispensed onto a 384-pillar plate. We describe automated cell-matrix dispensing, calcein-AM viability staining, and high-content fluorescence imaging for IC_50_ analysis. This protocol enables scalable, standardized, and high-throughput drug screening with tumor organoids for quantitative drug efficacy evaluation.

## Highlights

• Steps for establishing robust and scalable 3D organoid models using 384-pillar plates
• Instructions for automated embedding of SKOV-3 organoids in growth-factor-reduced Matrigel
• Guidance on automating compound dispensing and IC_50_ analysis in 384-well plates
• Steps for assessing cell viability using calcein-AM staining and high-content imaging

## Abstract (editor highlights)

Publisher’s note: Undertaking any experimental protocol requires adherence to local institutional guidelines for laboratory safety and ethics.

## Graphical abstract

**Figure:**
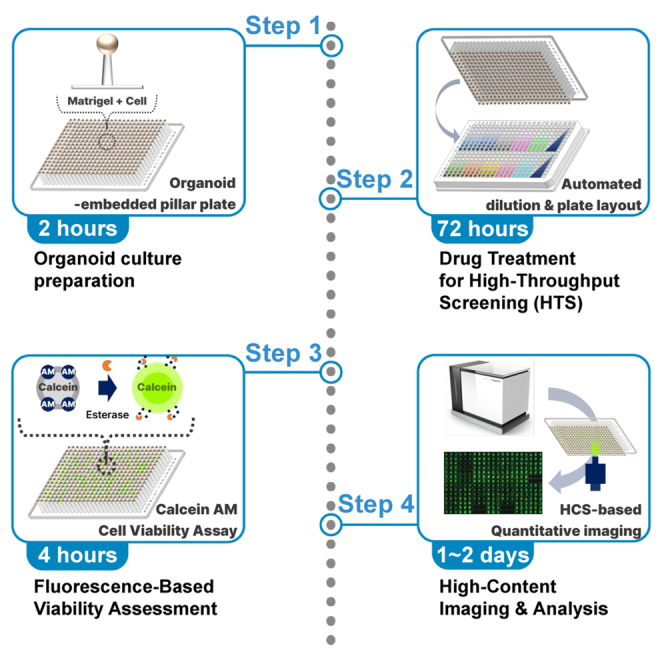

## Before you begin

Tumors are composed of spatially complex and heterogeneously organized cellular populations, creating a microenvironment that significantly influences cell behavior, drug response, and therapeutic efficacy.^1^^,^^2^^,^^3^ Traditional two-dimensional (2D) cell culture models, while simple and reproducible, have inherent limitations in recapitulating the structural organization and microenvironmental interactions observed in tumors.^4^^,^^5^ These limitations ultimately weaken the predictive value of drug screening and therapeutic evaluations.^6^^,^^7^

To overcome these limitations, three-dimensional (3D) culture systems, such as spheroids and organoids, have emerged as more physiologically relevant in vitro models.^8^^,^^9^^,^^10^ These systems better mimic the tumor microenvironment, enabling more accurate prediction of in vivo drug behavior and supporting translational cancer research.^11^^,^^12^^,^^13^ Consequently, in response to this development, there is an increasing need to develop 3D organoid-based high-throughput screening (HTS) platforms, which can facilitate large-scale and more accurate drug screening, offering greater potential for precision medicine and drug discovery in cancer research.^14^^,^^15^^,^^16^^,^^17^

However, conventional dome-based 3D cultures remain constrained by technical challenges that limit their scalability and automation compatibility, including variability in dome size, high reagent consumption, and difficulty in handling viscous matrices. To address these limitations, we developed a miniaturized 3D organoid culture platform compatible with automated liquid handling and high-content imaging systems for scalable drug screening. The following protocol outlines this approach using a vertical 384-pillar plate format.

### Innovation

This protocol introduces a vertical 384-pillar plate platform that enables gravity-assisted, high-precision deposition of high-viscosity Matrigel–cell mixtures at the microliter scale. Unlike conventional dome-based 3D cultures, which suffer from variability in dome size, high reagent consumption, and limited automation compatibility, this platform allows precise and reproducible dispensing of 1.5 μL Matrigel–cell droplets in a highly scalable format. By utilizing gravity-assisted placement and automation-compatible plate design, it achieves uniform spheroid formation, improved screening consistency, and efficient reagent use. The miniaturized pillar-array system maintains the physiological relevance of 3D tumor models while enhancing throughput and reproducibility. Integration with Calcein-AM–based viability staining and high-content fluorescence imaging enables quantitative drug efficacy assessment through IC_50_ analysis. Taken together, this workflow bridges the gap between biological complexity and operational efficiency, providing a standardized and automation-ready platform for high-throughput organoid-based drug screening and precision oncology research.

### Preparation of routine cell culture of SKOV-3 cells

**Timing: 2–3 days**

SKOV-3 cells should be maintained under standard 2D culture conditions prior to experimental use. This section describes how to thaw, expand, and subculture SKOV-3 cells.

1. Thaw frozen SKOV-3 cells in a 37°C water bath for 1–2 minutes until a small ice crystal remains.
2. Transfer 5.0 × 10^5^ cells per flask to 9 mL of pre-warmed complete McCoy’s 5A medium (supplemented with 10% FBS and 1% penicillin-streptomycin).
  a. Culture medium: 445 mL base medium + 50 mL FBS (10%) + 5 mL penicillin-streptomycin (1%).
3. Wash cells once to remove DMSO and plate in T25 or T75 flasks.
4. Incubate at 37°C with 5% CO_2_, changing medium every 2–3 days.
5. Subculture when confluency reaches ∼80%–90% using 0.25% Trypsin-EDTA.
  a. Aspirate the culture medium and rinse cells once with sterile PBS.
  b. Add 1 mL of 0.25% Trypsin-EDTA to cover the cell layer and incubate at 37°C for 3–5 min, monitoring under a microscope until most cells round up and detach.
  c. Neutralize the trypsin by adding 9 mL of pre-warmed complete McCoy’s 5A medium.
  d. Collect the cell suspension and centrifuge at ∼300 × g for 5 min.
  e. Carefully aspirate the supernatant and resuspend the cell pellet in fresh complete McCoy’s 5A medium by gentle pipetting.
  f. Transfer the resuspended cells into new T75 flasks at the desired seeding density.
6. Maintain cells below passage 30 and routinely test for mycoplasma contamination.

***Note:*** Always verify cell health under the microscope and perform mycoplasma testing routinely.

### Preparation of Matrigel handling

**Timing: ∼1 day**

Matrigel is a thermosensitive ECM hydrogel that rapidly polymerizes above 10°C. Proper handling is critical for consistent organoid morphology and imaging quality.

7. Thaw Matrigel slowly at 4°C overnight.
  a. Do not thaw at room temperature (20°C–25°C), as this leads to premature gelation.
8. On the day of use, maintain all Matrigel and related materials on ice, including pipettes, pipette tips, tubes, and plates.
9. Pre-cool all consumables and equipment (pipette tips, 384-pillar plates, microcentrifuge tubes) to 4°C before use.
10. Handle Matrigel slowly and gently using wide-bore or cut tips to avoid introducing air bubbles.

***Note:*** Matrigel rapidly solidifies at >10°C. Strict temperature control is essential for consistency and reproducibility.

**CRITICAL:** Avoid bubble formation when pipetting Matrigel. Bubbles can interfere with subsequent image acquisition and cell viability.

### Preparation of plate layout design

**Timing: ∼1 day**

Proper plate layout planning is essential for ensuring statistical robustness and minimizing variability during high-throughput screening.

11. Design a 384-well plate layout to screen pharmacological compounds per plate.
12. Each compound should be tested at seven concentrations in triplicate to support robust statistical analysis.
13. Avoid using wells in the outermost rows and columns of a 384-well plate (i.e., rows A and P, and columns 1 and 24), where edge effects commonly occur due to temperature gradients and evaporation.
14. Assign positive control wells using a cytotoxic reference compound (e.g., Staurosporine or Cisplatin) to induce reproducible viability loss, and negative control wells with untreated cells in the inner region of the plate to define the dynamic range for viability normalization.

***Note:*** Use the same plate layout across all biological and technical replicates to ensure comparability across experiments.

**CRITICAL:** Edge effects in the outermost rows and columns can lead to artificial variability in cell viability and fluorescence signals. Design your layout to exclude these wells and concentrate controls and test conditions within the central area.

### Preparation of screening libraries

**Timing: ∼1 day**

Before initiating organoid-based drug screening, compound libraries must be properly sourced, aliquoted, and stored to maintain chemical stability and ensure reproducibility across plates.

15. Obtain pharmacological compounds from commercial suppliers as 10 mM stock solutions dissolved in DMSO (e.g., TargetMol).
16. Aliquot stock solutions into low-binding polypropylene (PP) 96-well or 384-well plates to minimize compound loss due to surface adsorption.
17. Seal and store compound plates at −20°C for long-term storage (up to 6–12 months) and at 4°C for short-term use (up to 1–2 weeks). Minimize freeze–thaw cycles to preserve compound stability.
18. Thaw plates at room temperature (20°C–25°C) prior to use and mix gently before dispensing.

***Note:*** Avoid repeated freeze-thaw cycles. Use freshly thawed aliquots for each experiment to preserve compound integrity.

**CRITICAL:** Compounds dissolved in DMSO are highly sensitive to moisture and light. Work quickly and minimize exposure to ambient air and light during handling.

### Organoid pre-screening characterization

**Timing: Optional (∼1 day)**

Before initiating large-scale screening, perform a pilot test to determine optimal seeding density. This pre-screening step enables assessment of organoid growth kinetics and morphological uniformity, which improves reproducibility and ensures consistent organoid formation for HTS.

19. Embed organoids with varying initial cell densities (e.g., 500, 1000, 2000 cells per pillar) using the standard Matrigel-embedding protocol.
20. Monitor organoid viability and morphology over a 1–3 day period using fluorescence imaging (e.g., Calcein-AM staining).
21. Compare growth kinetics and structural consistency to select a seeding density that yields uniform spheroids suitable for HTS.

***Note:*** This optimization step enables the generation of organoids with consistent morphology and viability, thereby maximizing screening reproducibility and data robustness.

**CRITICAL:** Avoid selecting a seeding density that results in overly compact or necrotic spheroids, as it can introduce variability in drug penetration and response.

## Key resources table

**Table:** 

REAGENT or RESOURCE	SOURCE	IDENTIFIER
**Chemicals, peptides, and recombinant proteins**		

McCay’s 5A medium	Welgene	LM005-01
Fetal Bovine Serum	Gibco	16000044
Penicillin-Streptomycin	Sigma	P4458
Grown Factor Reduced Basement Membrane Matrix	Corning	354230
Paclitaxel	Sigma	T7402
Carboplatin	Sigma	C2538
Staurosporine	Sigma	S-4400
Calcein-AM	Invitrogen	C1430
PBS (10X), pH 7.4	Gibco	70011–069
0.25% Trypsin-EDTA	Thermo Fisher Scientific	25200056
DPBS	Thermo Fisher Scientific	14190144
DMSO	Sigma	D4540
Erlotinib HCl	Selleckchem	S1023
NVP-BKM120	Targetmol	T1827
AZD-1480	MedChemExpress	HY-10193
Lapatinib ditosylate monohydrate	Targetmol	T0078L
Olaparib	Targetmol	T3015
Ruxolitinib (INCB-18424) phosphate	Targetmol	T3043
Crizotinib	Targetmol	T1661
Bleomycin sulfate	Targetmol	T6116
ALK5 Inhibitor IV	Targetmol	T3031
Canertinib dihydrochloride	Targetmol	T2501
NVP-BAW2881	Targetmol	T3641
Osimertinib mesylate	Targetmol	T3634
BGP15	Targetmol	T3649
(Rac)-SAR131675	Targetmol	T3691
SU11274	Targetmol	T6154
Degrasyn	Targetmol	T6300
PF477736	Targetmol	T6028
Onatasertib	Targetmol	T3351
SUN 11602	Targetmol	T3714
Tipifarnib	Targetmol	T6271
Niraparib	Targetmol	T3231
GNF-5837	Targetmol	T6097
Allitinib tosylate	Targetmol	T6331
WZ4002	Targetmol	T6238
Itacitinib	Targetmol	T3998
Vactosertib	Targetmol	T6496
RG14620	Targetmol	T3554
Nazartinib	Targetmol	T3506
FIIN-2	Targetmol	T6836
Src Inhibitor 1	Targetmol	T3593
Desmethylanethol trithione	Targetmol	T3560
PD-166866	Targetmol	T3492
PD-168393	Targetmol	T6932
NVP-ACC789	Targetmol	T3463
SU6656	Targetmol	T6997
Telatinib	Targetmol	T6166
CHIR-124	Targetmol	T6350
NVP-AEW541	Targetmol	T6080
PHA-665752	Targetmol	T6128
PX-478	Targetmol	T6961
Bafetinib	Targetmol	T6311
MGCD-265 analog	Targetmol	T6351
Tirbanibulin	Targetmol	T6345
JI101	Targetmol	T3476
CP-724714	Targetmol	T4014
Anlotinib Dihydrochloride	Targetmol	T4094
SAR-20347	Targetmol	T4210
TAK-659 hydrochloride	Targetmol	T4209
SGI-7079	Targetmol	T6982
Bisantrene	Targetmol	T4047
Prexasertib dihydrochloride	Targetmol	T4327
Rucaparib	Targetmol	T4463
Linrodostat	Targetmol	T4532
ME0328	Targetmol	T6578
Paraxanthine	Targetmol	T4973
CD73-IN-3	Targetmol	T8875

**Experimental models: Cell lines**		

SKOV-3 human ovarian adenocarcinoma cell line	ATCC	HTB-77

**Software and algorithms**		

GraphPad Prism7	GraphPad	Prism Windows 7

**Other**		

Cellvitro 384MC Plate	MBD	MBD-CP-MC384B
Non-Contact Dispensing System	MBD	ASFA^Ⓡ^ SPOTTER
High-Content Imaging System	MBD	ASFA^Ⓡ^ SCANNER

## Materials and equipment

**Table:** SKOV-3 organoid culture medium

Reagent	Final concentration	Amount
McCay’s 5A medium	N/A	445 mL
Fetal Bovine Serum	N/A	50 mL
Penicillin-Streptomycin (5,000 Unit, 5 mg/mL)	50U/mL and 50 μg/mL	5 mL
**Total**	**N/A**	**500 mL**

Once prepared, keep at 4°C for up to 2 weeks.

**Table:** Staining reagents

Reagent	Final concentration	Amount
Calcein-AM (1 mM/DMSO)	4 μM	68 μL
PBS	N/A	16,932 μL
**Total**	**N/A**	17,000 μL

Once prepared, use entire volume immediately for one 384-well plate.

## Step-by-step method details

### Organoid culture preparation

**Timing: ∼2 h (excluding 24 h incubation)**

This step describes the entire process of organoid culture preparation using the 384-pillar plate system. It includes harvesting SKOV-3 cells, embedding them in Matrigel, dispensing onto pillar plates, polymerizing the matrix, and initiating organoid formation through a 24-hour incubation. This foundational setup enables downstream drug treatment and high-content imaging.

1. Experimental Setup.
  a. Set laboratory temperature to 22°C–25°C and pre-warm CO_2_ incubators to 37°C.
  b. Pre-cool empty 384-well plates at 4°C before use.
  c. Thaw Matrigel at 4°C and mix thoroughly before use.
  d. Pre-warm both an empty 384-well plate and a 384-well plate filled with medium in a CO_2_ incubator at 37°C.

***Note:*** Matrigel is liquid at 4°C but solidifies at 37°C. Proper thawing ensures consistent viscosity and usability. Store Matrigel at −70°C and transfer to 4°C for slow thawing 1 day before use.

2. Cell Culturing and Harvesting SKOV-3 cells.
  a. Check the cell culture confluency.
    i. Culture SKOV-3 cells in a T-75 flask with McCoy’s 5A medium supplemented with 10% fetal bovine serum (FBS) and 1% penicillin-streptomycin.
    ii. Incubate at 37°C in a humidified atmosphere with 5% CO_2_.
    iii. When the cells reach ∼80%–90% confluency, proceed to the next step.
  b. Remove the spent medium.
    i. Aspirate the culture medium completely using a vacuum line or serological pipette.
  c. Wash the cells.
    i. Gently rinse the monolayer once with 5 mL of pre-warmed phosphate-buffered saline (PBS) without Ca^2+^ and Mg^2+^.
    ii. Tilt and gently rotate to remove residual serum, which may inhibit trypsin activity.
  d. Add trypsin to detach the cells.
    i. Add 1 mL of 0.25% Trypsin-EDTA to the flask.
    ii. Gently tilt the flask to evenly distribute the trypsin.
    iii. Incubate at 37°C for 2–3 minutes, monitoring under a microscope for cell detachment.
    iv. Tap the side of the flask lightly to facilitate detachment if needed.
      ***Note:*** Do not over-trypsinize the cells. Overexposure can reduce cell viability and affect downstream aggregation or organoid formation.
  e. Neutralize the trypsin.
    i. Add 9 mL of complete McCoy’s 5A medium to stop trypsin activity.
    ii. Gently pipette up and down 5–10 times using a 10 mL pipette to obtain a single-cell suspension.
  f. Transfer cells to a 15 mL conical tube.
    i. Centrifuge at 300 × g for 3 minutes at room temperature (20°C–25°C).
    ii. Carefully aspirate the supernatant without disturbing the pellet.
  g. Resuspend the cell pellet.
    i. Add 10 mL of McCoy’s 5A medium and gently pipette to resuspend the cells.
  h. Count the cells.
    i. Use a hemocytometer or automated cell counter with trypan blue staining to assess cell viability.
    ii. Ensure cell viability is >90% before proceeding. If viability is below 90%, discard the cells and prepare a fresh culture before starting the experiment.
  i. Adjust the final cell concentration.
    i. Dilute the cell suspension with culture medium to achieve a final concentration of 1.0 × 10^3^ cells per 1.5 μL, equivalent to 6.67 × 10^5^ cells/mL.
    ii. Keep the cell suspension on ice or at room temperature (20°C–25°C) until use (within 30 minutes).
3. Embedding Cells in Matrigel and Forming 3D Organoids.
  a. Prepare Matrigel and cell suspension.
    i. Thaw growth factor-reduced Matrigel (Corning #354230) on ice overnight at 4°C.
    ii. Maintain all pipette tips, tubes, and reagents on ice to prevent premature gelation.
    iii. Adjust the concentration of SKOV-3 cells to 6.67 × 10^5^ cells/mL, as prepared in Step 2.
  b. Mix cells with Matrigel at a 3:7 ratio (v/v).
    i. In a pre-chilled 1.5 mL microcentrifuge tube, mix with 30 μL of cell suspension and 70 μL of cold Matrigel.
    ii. Use wide-bore or cut pipette tips to gently mix by slow pipetting (5–6 times).
      ***Note:*** Always prepare slightly more mixture (∼10%) to account for dead volume loss.
      **CRITICAL:** Avoid introducing bubbles during mixing, as this can interfere with organoid formation and downstream analysis.
  c. Dispense 1.5 μL of the cell–Matrigel mixture onto each pillar.
    i. Pre-cool the 384-pillar plate on ice or in a 4°C cold block for at least 10 minutes.
    ii. Using a pre-chilled automated low-volume dispenser (e.g., ASFAⓇ SPOTTER), dispense 1.5 μL of the mixture onto the center of each pillar.
    iii. Pre-cool all pipettes, tips, and 384-pillar plates at 4°C before use.
      **CRITICAL:** Matrigel polymerizes rapidly above 10°C. All reagents, plates, and pipette tips must be kept on ice. Premature gelation will result in uneven organoid morphology and size variation across the plate.
  d. Center cells by gravity.
    i. Keep the plate at 4°C for 10 minutes to allow cells to settle at the bottom of the Matrigel droplet (center of gravity).
    ii. Do not disturb the plate during this step.
      ***Note:*** Do not shake or disturb the plate during this 4°C incubation, or cell centering will be uneven.
  e. Polymerize the Matrigel at 37°C.
    i. Equilibrate the plate at room temperature (20°C–25°C) for 10–15 minutes before incubation to prevent condensation on the pillar surface.
    ii. Transfer the plate to a 37°C incubator for 40 minutes to allow complete Matrigel polymerization.
    iii. Avoid airflow disturbance; polymerization is sensitive to vibration and humidity.
      **CRITICAL:** Do not transfer the plate directly from 4°C to 37°C, as this can cause condensation and interfere with Matrigel solidification.
      **CRITICAL:** Ensure the incubator is placed on a flat, vibration-free surface. Any tilting may cause asymmetrical Matrigel solidification, affecting organoid uniformity.
  f. Mount the pillar plate onto a receiver 384-well plate.
    i. Pre-warm 60 μL of complete McCoy’s 5A medium into each well of a matching 384-well receiver plate (deep well U-bottom preferred).
    ii. Carefully align and mount the polymerized 384-pillar plate onto the receiver plate.
    iii. Press gently until contact is uniform.
      ***Note:*** Carefully align the pillar and receiver plates to maintain uniform contact and prevent uneven diffusion of nutrients and compounds.
  g. Incubate organoids before drug treatment.
    i. Incubate the assembled plate for 24 h at 37°C, 5% CO_2_ to allow spheroid formation and baseline organoid maturation.
    ii. Do not shake or move the plate during this incubation to ensure uniform organoid shape and location.
      ***Note:*** Maintain the plate in a stable, undisturbed condition for 24 h to support uniform spheroid growth.
  h. Assess organoid formation reproducibility.
    i. After 24 h incubation, acquire fluorescent or bright-field images of organoids using a high-content imaging system.
    ii. Discard wells with abnormal signal intensity, edge effects, or segmentation artifacts during image processing.
      ***Note:*** Wells were excluded under the following conditions: edge effects (rows A/P; columns 1/24), absence of organoid formation, instrumental noise (abnormal fluorescence spikes or imaging artifacts), or non-spherical morphology (fragmented or dumbbell-shaped organoids).
    iii. Calculate the coefficient of variation (CV%) across replicate wells to evaluate uniformity of organoid formation.
    iv. Exclude plates with CV > 10% or poor morphology from downstream analysis.
      ***Note:*** Reproducibility assessment prior to compound treatment ensures data reliability in screening.
      **CRITICAL:** Inconsistently formed organoids compromise assay reproducibility and dynamic range.

### Drug treatment and screening

**Timing: ∼72 h (including incubation)**

This step covers the preparation of serially diluted drug plates and treatment of Matrigel-embedded organoids using the 384-pillar plate format.

4. Prepare drug plates with serial dilutions (e.g., 2-fold dilutions from 100 μM).
  a. Prepare 100 μM working solutions in 0.1% DMSO by diluting the 10 mM stock solutions in DMSO with assay medium.
  b. Perform 2-fold serial dilutions across designated wells of a 384-well deep-well plate using an automated liquid handling system (e.g., Tecan) or a manual multichannel pipette.

***Note:*** Prepare at least 10% excess volume to compensate for dead volume during dispensing.

***Note:*** Use the predefined plate layout (see before you begin) to ensure consistent placement of compounds, controls, and blanks across replicates.

5. Ensure DMSO concentration is uniform (typically 0.1%) across all wells.
  a. Adjust all wells so that the final DMSO concentration is consistent (e.g., 0.1%) across drug-treated and control wells.
  b. Mix gently by pipetting to ensure uniform drug distribution.

**CRITICAL:** Variability in DMSO concentration can confound dose-response outcomes and induce cytotoxicity unrelated to the compound of interest.

6. Attach the organoid-loaded pillar plate to the drug-containing 384-well plate.
  a. Retrieve the organoid-embedded 384-pillar plate that has been incubated for 24 h (Step 3g).
  b. Carefully align and mount the pillar plate onto the drug-containing 384-well plate.
  c. Press gently and uniformly to ensure full contact between Matrigel droplets and drug-containing medium.

***Note:*** Use visual inspection to confirm there are no air bubbles trapped between pillars and wells. If air bubbles are observed during visual inspection, gently re-seat the pillar plate or lightly tap the plate to dislodge them. Plates with persistent bubbles should be discarded and re-prepared, as bubbles interfere with drug diffusion and organoid growth.

7. Incubate for 72 hours at 37°C with 5% CO_2_.
  a. Incubate the plate assembly at 37°C with 5% CO_2_ for 72 h.
  b. Avoid plate disturbance during the incubation period to maintain consistent organoid-drug interactions.

**CRITICAL:** Incubation beyond 72 h may lead to nutrient depletion or changes in drug stability, impacting viability readouts.

### Cell viability analysis using calcein-AM

**Timing: ∼4 h (including incubation and washing)**

This step describes the assessment of organoid viability following drug treatment using Calcein-AM, a fluorescent esterase substrate that stains live cells green. The signal is detected using an automated fluorescence microscope equipped with a FITC filter.

8. Prepare 4 μM Calcein-AM in pre-warmed PBS.
  a. Prepare a 1 mM stock solution of Calcein-AM in DMSO. Store at −20°C protected from light.
  b. Immediately before use, dilute the stock in pre-warmed PBS (37°C) to a final working concentration of 4 μM.
  c. Mix gently without vortexing.

***Note:*** Calcein-AM is light-sensitive; prepare solution under low light and use within 30 min.

9. Stain organoids with Calcein-AM.
  a. After the 72 h drug incubation (Step 4), detach the organoid-loaded pillar plate from the drug plate.
  b. Place the pillar plate onto a fresh 384-well plate containing 40 μL/well of the 4 μM Calcein-AM solution.
  c. Incubate for 1 h at 37°C, 5% CO_2_.

**CRITICAL:** Avoid shaking or disturbing the plate during incubation, as movement can affect dye penetration and organoid staining uniformity.

10. Wash organoids three times with PBS (15 minutes each).
  a. Transfer the pillar plate sequentially to three fresh 384-well plates, each filled with 60 μL of PBS per well.
  b. Allow passive diffusion of dye into PBS for 15 min per wash at room temperature (20°C–25°C).
  c. Avoid pipetting or agitation to prevent damage to Matrigel structure.

***Note:*** Gentle passive diffusion is preferred over active washing to preserve 3D organoid integrity.

11. Acquire fluorescence images.
  a. Mount the stained pillar plate on an imaging-compatible 384-well receiver plate containing 60 μL PBS per well.
  b. Image using an automated fluorescence microscope (e.g., ASFA SCANNER) equipped with a FITC/GFP filter set (Ex: ∼488 nm, Em: ∼520 nm).
  c. Set imaging parameters:
    i. Shutter speed: 15 ms.
    ii. Gain: 2.
    iii. Objective: 4× or 10× depending on organoid size.
  d. Acquire images suitable for the organoid depth.

***Note:*** If using an imaging system capable of Z-stack acquisition, 3D spheroidal morphology of organoids can be observed, providing spatial context beyond single-plane imaging.

**CRITICAL:** Consistent imaging parameters across all wells and plates are essential for quantitative comparison. Avoid photobleaching by minimizing exposure.

### Data analysis and interpretation

**Timing: ∼1–2 days (depending on image volume and processing workflow)**

This step outlines the process for analyzing high-content imaging data from organoid-based screening. Fluorescence intensity values are extracted, normalized, and used to generate dose–response curves for compound efficacy evaluation.

12. Analyze acquired images.
  a. Import fluorescence images into analysis software (e.g., ASFA SCANNER, ImageJ, or equivalent high-content image analysis platform).
  b. Review image quality to exclude wells with edge effects, low signal, or bubble artifacts.
13. Segment organoids and quantify fluorescence intensity.
  a. Apply the automated segmentation function of the analysis software (e.g., ASFA SCANNER or equivalent) to identify individual organoids within each well.
  b. Measure total or average fluorescence intensity (e.g., Calcein-AM signal) per organoid.
  c. Export raw intensity data for all wells into a spreadsheet or compatible file format.
14. Normalize fluorescence data using controls.
  a. Set the average intensity of negative control wells (media only) as 0% viability.
  b. Set the average intensity of positive control wells (untreated cells) as 100% viability.
  c. Calculate the relative viability of each drug-treated well as a percentage of the control range.
15. Generate dose–response curves.
  a. Import normalized viability data into GraphPad Prism or similar software.
  b. Plot relative cell viability (% of mock) vs. concentration of screening compound.
  c. Fit a nonlinear regression model (e.g., variable slope sigmoidal dose–response) to each compound.
16. Calculate IC_50_ values.
  a. Determine the concentration of compound that reduces viability by 50% (IC_50_) from the fitted curves.
  b. Export IC_50_ values and confidence intervals for comparison between compounds.

## Expected outcomes

**Figure 1:**
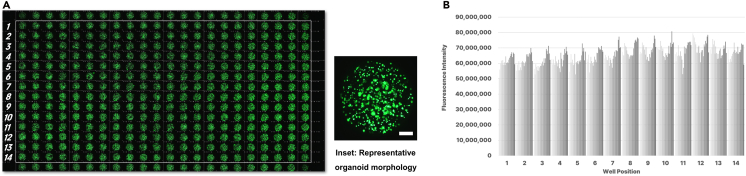
Assessment of organoid viability and assay uniformity in a 384-pillar plate format (A) Representative whole-plate fluorescence image of SKOV-3 ovarian cancer organoids stained with Calcein-AM after three days of culture. Green fluorescence indicates viable cells metabolically converting Calcein-AM via intracellular esterase activity. Organoids were seeded at 1,000 cells per pillar and cultured under standardized conditions. *Inset*: Representative organoid morphology (SKOV-3 cell-derived 3D structure; scale bar, 400 μm). (B) Quantitative analysis of fluorescence intensity across selected wells. To avoid edge effects, only wells in columns 1–14 and row 22 (total n = 308) were analyzed. Bar graph shows fluorescence intensity distribution per well. The coefficient of variation (CV) was calculated to be 8.5%, indicating high reproducibility and assay reliability in this miniaturized high-throughput format.

This protocol enables high-throughput, fluorescence-based assessment of cytotoxicity and proliferation in three-dimensional (3D) organoids derived from the SKOV-3 ovarian cancer cell line. Approximately 2,000-μm-diameter organoids were generated by seeding 1,000 SKOV-3 cells per pillar and culturing for three days in the 384-pillar plate format. Viable cells were stained using Calcein-AM, and fluorescence intensity was quantified using a high-content imaging system. The coefficient of variation (CV) of fluorescence signals across wells was 8.5%, demonstrating the high reproducibility and reliability of the system under standardized assay conditions (Figure 1).

**Figure 2:**
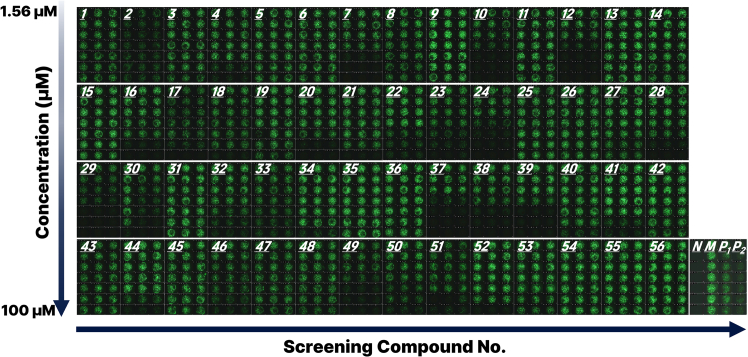
Representative fluorescence microscopy images of SKOV-3 organoids following compound treatment Three-dimensional SKOV-3 ovarian cancer organoids were exposed to a panel of 56 small-molecule compounds across a concentration gradient. Each panel (1–56) corresponds to a distinct compound, with drug concentrations increasing from top (1.56 μM) to bottom (100 μM) within each panel. Green fluorescence signals were derived from intracellular conversion of Calcein-AM, a viability-sensitive dye activated by cellular esterase activity in live cells. Images were obtained from three independent replicates (n = 3) and present qualitative differences in viability responses elicited by compound treatment.

Drug screening was performed using 56 small-molecule compounds related to ovarian cancer signaling, apoptosis, and metabolism (Table S1). Each of the 56 compounds was tested in a seven-point serial dilution starting from 100 μM, and organoids were exposed for 72 h. Control groups included N (negative control; no cells, media only), M (mock-treated; media only with cells), P_1_ (positive control; staurosporine, 2.5 μM), and P_2_ (positive control; carboplatin, 100 μM + paclitaxel, 10 μM). Fluorescence imaging based on Calcein-AM staining provided qualitative insights into the viability of organoids, as shown in Figure 2. The compound panel exhibited diverse cellular responses, including strong, moderate, and minimal cytotoxic effects. Several compounds demonstrated clear dose-dependent antiproliferative activity. The responses of the positive controls confirmed that the platform is sufficiently sensitive to detect viability reduction induced by both cytotoxic (staurosporine) and cytostatic (carboplatin + paclitaxel) agents, supporting its utility for functional drug screening.

**Figure 3:**
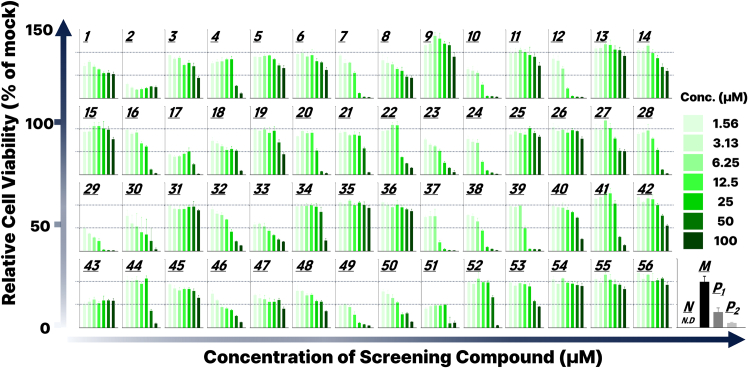
Quantitative analysis of organoid viability based on fluorescence intensity Relative cell viability of SKOV-3 organoids was quantified from the fluorescence images shown in Figure 2 using image analysis software. Fluorescence intensity values were normalized to the mock-treated control (100 % viability) and the negative control (0 % viability) as defined in Table S1. Data are presented as mean ± standard deviation from three biological replicates (n = 3) for each compound and concentration. In each bar graph, increasing compound concentrations are represented by increasing color intensity, as indicated in the color legend. This visual encoding enables intuitive interpretation of dose-dependent viability responses across the compound panel.

**Figure 4:**
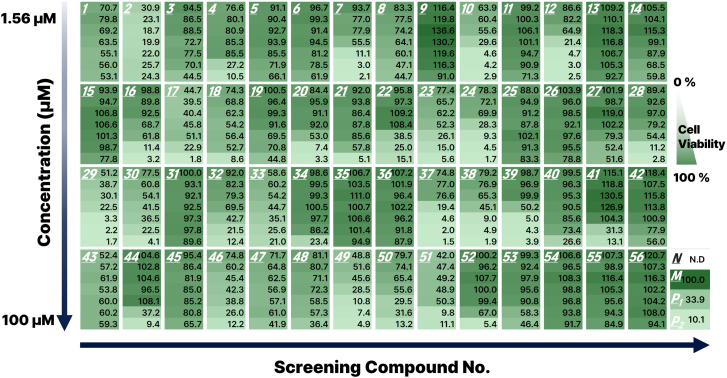
Heatmap visualization of compound-induced viability profiles A heatmap was constructed using the normalized cell viability data from Figure 3 to enable comparative visualization of cytotoxic responses across the entire compound panel. Each row represents a distinct compound (1–56), and each column corresponds to a specific concentration. Within each panel, compound concentration increases from top (1.56 μM) to bottom (100 μM). Color intensity reflects relative cell viability, with lower viability indicated by brighter hues (0 %) and higher viability by darker hues (100 %). This visualization enables rapid identification of dose-dependent patterns, including strongly cytotoxic, partially active, or non-responsive compounds.

Subsequent image-based quantification enabled robust numerical analysis of organoid viability, and the resulting data are presented in Figure 3. These quantified viability values were visualized in the form of a heatmap, facilitating intuitive comparison of compound efficacy and response patterns across the screening panel (Figure 4).

**Figure 5:**
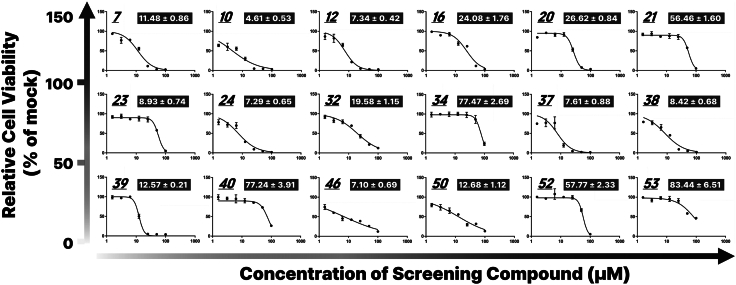
Nonlinear regression analysis of dose–response curves for selected compounds to determine IC_50_ values Compounds exhibiting clear dose-dependent inhibition of organoid viability were selected for pharmacodynamic modeling. Normalized viability data were fitted to a four-parameter logistic regression model to generate sigmoidal dose–response curves. Graphs present mean cell viability ± standard deviation (n = 3) at each concentration, overlaid with the corresponding fitted curve. IC_50_ values were calculated from the fitted model and are presented as mean ± standard error (SE) within each panel, allowing for comparative assessment of compound potency across the screened set.

Among the screened compounds, 18 that exhibited clear dose–response relationships were selected for IC_50_ determination. The calculated IC_50_ values ranged from 4.6 μM for Canertinib dihydrochloride (No. 10) to 83.4 μM for Linrodostat, confirming the platform’s capacity to quantitatively rank compound potency and prioritize anticancer candidates based on efficacy (Figure 5).

The miniaturized assay format minimizes sample and reagent consumption while allowing for the parallel evaluation of multiple drug conditions. The system is fully compatible with automated organoid culture workflows and high-content imaging platforms, offering a scalable, cost-effective solution for preclinical drug screening in ovarian cancer models and beyond.

## Quantification and statistical analysis

Fluorescence imaging-based screening data were obtained as shown in Figure 2. The analysis process consisted of three main steps: (1) image acquisition, (2) fluorescence intensity quantification, and (3) normalization relative to control conditions.

1. Image acquisition.

SKOV3 organoids stained with calcein-AM were cultured in a 384-well plate and imaged using high-content imaging system(ASFA^Ⓡ^ SCANNER). Wells with technical issues, including incomplete image acquisition, edge artifacts, or irregular growth patterns, were excluded from further analysis to ensure data accuracy and reproducibility.

2. Fluorescence intensity quantification.

Fluorescence signals were quantified using ASFA SCANNER. Image segmentation was applied to define the organoid area, and background fluorescence was subtracted to enhance signal specificity. Only wells with sufficient image coverage and quality were retained for analysis. The mean fluorescence intensity (FI) per well was used as the quantitative readout of cell viability.

3. Normalization and data processing.

Quantified fluorescence intensity (FI) values were normalized relative to the two control conditions: the negative control (FI_N_; media only, no cells), defined as 0% viability, and the mock-treated control (FI_M_; media only, with cells), defined as 100% viability. The relative viability for each sample well was calculated using the following equation:

$$Viability\left(\%\right)=\frac{\left({\text{FI}}_{Sample}-{\text{FI}}_{N}\right)}{\left({\text{FI}}_{M}-{\text{FI}}_{N}\right)}\times 100$$

Normalized viability data and corresponding drug concentrations (log-transformed) were exported to GraphPad Prism for nonlinear regression analysis. Dose–response curves were generated using the [log(inhibitor) vs. response – variable slope] option, and IC_50_ values were obtained directly from the regression output.

## Limitations

While this protocol enables efficient and reproducible high-throughput screening of cytotoxic and antiproliferative compounds using SKOV-3-derived 3D organoids, several limitations should be considered.

### Lack of tumor microenvironment complexity

The use of a monoclonal cancer cell line cultured in a simplified 3D matrix fails to replicate the complexity of the native tumor microenvironment.^13^ Essential components such as stromal cells, immune populations, and vasculature are absent, limiting the system’s utility for evaluating treatments that depend on tumor–host interactions, including immunotherapies and anti-angiogenic agents.^18^

### Inability to differentiate cytostatic and cytotoxic effects

The assay measures cell viability using calcein-AM fluorescence, which reflects intracellular esterase activity in live cells.^19^ Although this readout correlates with overall viability, it does not distinguish between cytostatic (growth-inhibitory) and cytotoxic (cell-killing) effects. Consequently, additional endpoint assays—such as apoptosis markers (e.g., caspase activity, Annexin V) or proliferation markers (e.g., Ki-67, EdU)—are recommended to gain mechanistic insight into drug responses.^20^^,^^21^

### Technical variability in fluorescence imaging and quantification

From a technical standpoint, fluorescence imaging and quantification in 384-well formats remain susceptible to environmental and mechanical variables. Factors such as uneven cell seeding, edge effects, photobleaching during acquisition, and inconsistencies in plate handling can cause variability in measurement accuracy.^22^ In addition, suboptimal image quality or heterogeneous staining may impair image segmentation, thereby reducing the accuracy of fluorescence-based quantification.^23^ These issues are particularly relevant in large-scale screens and should be closely monitored through appropriate quality control procedures.

## Troubleshooting

### Problem 1

Uneven Matrigel dome formation or bubble inclusion during cell seeding (Step 3c) can lead to inconsistent organoid growth and heterogeneity in drug exposure.

### Potential solution

To prevent uneven Matrigel domes or air bubbles during dispensing, it is critical to keep all components - including Matrigel, pipette tips, and reservoirs - pre-cooled at 4°C. Dispensing should be performed slowly and with wide-bore or cut tips to reduce mechanical stress and air introduction. Pre-chilling the 384-pillar plate using a cold block before seeding further supports uniform dome formation. Visual inspection immediately after seeding can help identify and discard any wells with visible bubbles.

### Problem 2

Cells do not settle properly at the base of the Matrigel dome during the pre-gelation step (Step 3d), resulting in off-center organoids that are difficult to image and analyze.

### Potential solution

To improve cell positioning within the Matrigel droplet, place the seeded pillar plate in a vibration-free 4°C environment for approximately 10 minutes. This pause allows gravity to guide cells toward the center of the droplet prior to gelation. Ensuring that the plate is perfectly level using a spirit level or flat bench surface further supports uniform cell settling.

### Problem 3

Formation of air gaps between the Matrigel dome and drug solution during plate mounting (Step 6b) can inhibit diffusion and impair treatment efficacy.

### Potential solution

Before mounting, confirm that each well of the drug plate contains a uniform volume of media (typically 60 μL). Slowly lower the pillar plate onto the drug plate and apply gentle, even pressure to ensure contact. If minor air gaps persist, a short centrifugation step (e.g., 300 rpm for 1 minute) can help eliminate trapped bubbles and improve media contact.

### Problem 4

Weak or uneven Calcein-AM fluorescence after staining (Step 9c) reduces signal-to-noise ratio and limits accurate viability assessment.

### Potential solution

Prepare Calcein-AM staining solution fresh each time and protect it from light exposure. Ensure that incubation is carried out at 37°C in 5% CO_2_ for 1 hour without disturbing the plate. Movement during staining can create uneven dye distribution or affect penetration into the organoid structure. If signal remains weak, validate dye concentration and confirm cell viability with a positive control.

### Problem 5

Organoids become damaged or displaced during PBS washing steps (Step 10), compromising morphology and measurement accuracy.

### Potential solution

To avoid disruption of the Matrigel structure, use only passive diffusion for washing steps. Transfer the pillar plate onto fresh PBS-filled receiver plates and allow a minimum of 15 minutes per wash step at room temperature (20°C–25°C). Avoid any pipetting, tilting, or shaking during the washing process, and minimize total handling time to maintain organoid integrity.

### Problem 6

High well-to-well variability in fluorescence signals or poor reproducibility in dose-response analysis (Step 14).

### Potential solution

Variability in IC_50_ curves or fluorescence data is often caused by inconsistencies in seeding density or edge effects. Prior to screening, optimize seeding density using a pilot study and use symmetric plate layouts to reduce perimeter-based drift. During analysis, exclude any wells with visual artifacts, signal loss, or bubbles. A coefficient of variation (CV%) of less than 10% across replicate wells is generally considered acceptable for high-content imaging–based assays.

## Resource availability

### Lead contact

Further information and requests for resources and reagents should be directed to and will be fulfilled by the lead contact, Min Sang Lee (ms_lee@gbsa.or.kr).

### Technical contact

Technical questions on executing this protocol should be directed to and will be answered by the technical contact, Jung Sook Choi (choijs@gbsa.or.kr).

### Materials availability

This protocol did not generate new unique reagents.

### Data and code availability

This paper did not generate new datasets.

## Acknowledgments

This research was supported by Development Project for Emerging Research Instruments Technology through the Commercialization Promotion Agency for R&D Outcomes (COMPA) grant funded by the Ministry of Science and ICT (MSIT) (2024-22030007-00 and 2710069214).

## Author contributions

J.S.C. and M.S.L. developed and optimized the protocol, conducted experiments, and wrote the manuscript. M.J.S. prepared and provided the screening compounds. K.J. supervised the project and revised the manuscript.

## Declaration of interests

The authors declare no competing interests.
