# Supplementary material for: Protocol for high-content drug screening using tumor organoids on a 384-pillar plate platform
Source: STAR Protoc. 2025 Dec 1;6(4):104250. doi: 10.1016/j.xpro.2025.104250 (PMC12718461; doi:10.1016/j.xpro.2025.104250)
Supplement: Document S1. Table S1 [file mmc1.pdf]

Table S1. **List of 56 small-molecule compounds and control groups used for viability screening, related to Expected Outcomes.** A total of 56 compounds related to ovarian cancer signaling, apoptosis, and metabolism were selected for high-throughput screening in SKOV-3 3D organoids. Each compound is assigned a unique identification number (No. 1–56), which is consistently used throughout all figures and analyses to ensure traceability. Compounds were tested in a seven-point dose–response format (starting at 100 μM) to evaluate their cytotoxic or cytostatic effects. Control groups included N (negative control; media only, no cells), M (mock-treated control; media only with cells), P_1 (positive control; carboplatin 100 μM + paclitaxel 10 μM), and P_2 (positive control; staurosporine 2.5 μM).

No.	Screening compound	No.	Screening compound
1	Desmethylanethol trithione	31	TAK-659 hydrochloride
2	ALK5 Inhibitor IV	32	RG 14620
3	Vactosertib	33	PX-478 2HCl
4	Degrasyn	34	Linrodostat
5	PHA665752	35	Ruxolitinib (INCB-18424) phosphate
6	BAW2881	36	Itacitinib
7	Bafetinib	37	SAR20347
8	SU11274	38	LY-3475070
9	PF477736	39	Niraparib
10	CC223	40	Olaparib
11	CHIR124	41	Rucaparib
12	Prexasertib dihydrochloride	42	ME0328
13	GNF5837	43	BGP15
14	Telatinib	44	Src Inhibitor 1
15	SGI7079	45	SU6656
16	MGCD-265 analog	46	KX2-391
17	Bleomycin Sulfate	47	Bisantrene
18	Osimertinib mesylate	48	Tipifarnib
19	Canertinib dihydrochloride	49	SAR131675
20	WZ4002	50	NVP-ACC789
21	Nazartinib	51	Anlotinib Dihydrochloride
22	CP724714	52	AZD-1480
23	PD168393	53	Crizotinib
24	AST-1306 TsOH	54	NVP-BKM120
25	1,7-DIMETHYLXANTHINE	55	Erlotinib HCl

26	JI101	56	Lapatinib ditosylate monohydrate
27	SUN 11602	N	Media only (no cells)
28	FIIN2	M	Media only with cells (100% viability)
29	PD166866	P_1	Carboplatin 100 μM + Paclitaxel 10 μM
30	NVP-AEW541	P_2	Staurosporine 2.5 μM
